# Removing the Clitoris in Cases of Masturbation Accompanied with Threatning Insanity

**Published:** 1862-07

**Authors:** E. S. Cooper

**Affiliations:** Professor of Anatomy and Surgery in the Medical Department of the University of the Pacific


*Removing the Clitoris in Cases of Masturbation accompanied with Threatning Insanity.* By E. S. Cooper, A. M. M. D., *Professor of Anatomy and Surgery in the Medical Department of the University of the Pacific.*

The proper management of inveterate cases of Masturbation, in both sexes, is a subject of great importance to medical men, in view of the fact of the practice causing a degeneration of intellect, and even insanity itself. These cases are far more numerous, in every country, than they are supposed to be, except by medical men. Being subjects of great delicacy, they remain, as they should be, secrets between the family and medical adviser. Though these are matters of a very private nature, they should always be freely discussed by medical men the more so, since very little has, thus far, been done to ameliorate the condition of the more unfortunate victims of this practice, who have progressed towards approaching insanity. But to the inexpressible anguish and chagrin of their parents and the most profound pity of all who know anything of them, they often degenerate, physically, morally and intellectually, until they pass out of the reach of medical aid, and become hopelessly lost. At no time does the practitioner feel more keenly the inability of his art to save, however often he may have to deplore his inefficiency in arresting the progress of some fatal malady. Death is infinitely preferable to insanity from this cause.

In the female, it would appear, judging from the result of two cases, as though we have found a remedy by surgical interference, and of such mildness and simplicity as to commend it to an early trial in all cases where the habit of Masturbation threatens injury of the intellect. It consists in removing the entire clitoris, including the corpus cavernosum clitoridis, and the major portion of the erectores clitoridis.

*Remarks.*—After these results, (referring to reported cases,) I think a surgeon should not only remove the clitoris, in all similar cases, but, if this should prove insufficient to effect a cure, he should remove the entire nymphæ, also, as they are very vascular, and possess erectile tissue of great sensibility, and are said to be parts often tiltillated in masturbation.

*Query.*—What effect will these operations have upon the person in the married state? These parts are supposed, by many physiologists, to have much influence over sensual gratification. Some regard them, in fact, as the seat of venereal pleasure.

Practically, this matter does not come up in those desperate cases in which a destruction of the mental faculties is rapidly going on, and there is no other known way of curing the patient. But, in a physiological point of view, it is a subject of great interest.—*San Francisco Medical Press.*

